# A Simple and Efficient Method for Visualizing Individual Cells *in vivo* by Cre-Mediated Single-Cell Labeling by Electroporation (CREMSCLE)

**DOI:** 10.3389/fncir.2020.00047

**Published:** 2020-07-28

**Authors:** Anne Schohl, Zahraa Chorghay, Edward S. Ruthazer

**Affiliations:** Department of Neurology and Neurosurgery, Montreal Neurological Institute-Hospital, McGill University, Montreal, QC, Canada

**Keywords:** transfection, morphology, neuron, multiphoton, loxP, *Xenopus laevis*, optic tectum

## Abstract

Efficient methods for visualizing cell morphology in the intact animal are of great benefit to the study of structural development in the nervous system. Quantitative analysis of the complex arborization patterns of brain cells informs cell-type classification, dissection of neuronal circuit wiring, and the elucidation of growth and plasticity mechanisms. Time-lapse single-cell morphological analysis requires labeling and imaging of single cells *in situ* without contamination from the ramified processes of other nearby cells. Here, using the *Xenopus laevis* optic tectum as a model system, we describe CRE-Mediated Single-Cell Labeling by Electroporation (CREMSCLE), a technique we developed based on bulk co-electroporation of Cre-dependent inducible expression vectors, together with very low concentrations of plasmid encoding Cre recombinase. This method offers efficient, sparse labeling in any brain area where bulk electroporation is possible. Unlike juxtacellular single-cell electroporation methods, CREMSCLE relies exclusively on the bulk electroporation technique, circumventing the need to precisely position a micropipette next to the target cell. Compared with viral transduction methods, it is fast and safe, generating high levels of expression within 24 h of introducing non-infectious plasmid DNA. In addition to increased efficiency of single-cell labeling, we confirm that CREMSCLE also allows for efficient co-expression of multiple gene products in the same cell. Furthermore, we demonstrate that this method is particularly well-suited for labeling immature neurons to follow their maturation over time. This approach therefore lends itself well to time-lapse morphological studies, particularly in the context of early neuronal development and under conditions that prevent more difficult visualized juxtacellular electroporation.

“Against a clear background stood black threadlets, some slender and smooth, some thick and thorny in a pattern punctuated by small dense spots. All was sharp as a sketch with Chinese ink on transparent Japanese paper. And to think that this was the same tissue which, when stained with carmine or logwood left the eye in a tangled thicket where sight may stare and grope ever fruitlessly, baffled in its efforts to unravel confusion, and lost forever in twilit doubt. Here, on the contrary, all was clear and plain as a diagram. A look was enough. Dumbfounded, I could not take my eyes from the microscope.”

Translation based on Santiago Ramon y Cajal’s “Histologia del Sistema Nervioso”, recounting his first encounter with Golgi-stained brain tissue (Glickstein, 2006).

## Introduction

The nature of the chaotic complexity of intermingled fibers and cellular structures in the nervous system constituted one of the great mysteries of anatomy, until Camillo Golgi’s serendipitous discovery of his “black reaction” in 1873 (Golgi, 1873). Golgi’s stain, which miraculously produced sparse, but highly contrasted labeling of cells in nervous tissue, prompted the brilliant Spanish neuroanatomist Santiago Ramon y Cajal to embrace the “neuron doctrine,” which states that individual brain cells, or neurons, are the fundamental units from which nervous tissue is formed (Jones, 2010).

Modern neuroanatomists have a far more diverse and powerful armamentarium at their disposal than Cajal might ever have imagined, including the use of genetically encoded labels like enhanced green fluorescent protein (EGFP) to visualize brain cells in the living organism. However, our efforts to dissect and decipher the nervous system continue to rely heavily on techniques that permit the labeling, observation and quantitative analysis of single cells within the richly complex network of brain cells and their elaborate processes from which neural circuits are constructed.

Single-cell electroporation (SCE) is a juxtacellular labeling technique that has been used successfully to transfect and visualize individual cells with Golgi-like contrast in intact brain tissue and in organotypic slice cultures (Haas et al., 2001; Uesaka et al., 2005). SCE is achieved by carefully positioning the fine (∼1 μm diameter) tip of a glass micropipette in close proximity to the cell of interest and then applying current pulses through the pipette tip. By locally disrupting the plasma membrane of the cell while iontophoretically expelling the charged contents of the micropipette, the contents of the pipette are efficiently delivered into the cell, which promptly reseals its plasma membrane and traps the material within.

Electroporation has numerous advantages compared to other transfection methods such as viral transduction. There is no strict size limitation for DNA constructs transferred by electroporation. The same cells can be electroporated repeatedly and a large number of different constructs can be electroporated into a cell at the same time, including the co-electroporation of fluorescent dye, mRNA, protein, or antisense oligonucleotides, together with plasmid DNA to permit immediate live cell imaging and manipulation. Moreover, most likely because of the high copy number of plasmids delivered, electroporation can drive high levels of expression of proteins such as EGFP within just a few hours of treatment, in many cases reaching peak levels within 1 day post-electroporation.

The main shortcoming of SCE is that successful labeling depends on the micropipette tip being positioned very precisely next to the targeted cell. If the tip is too far away it may transfect multiple nearby cells or fail to transfect any cells at all. Naturally, this is impractical in the majority of *in vivo* situations where the targeted cells are difficult to visualize under a microscope or so sparsely distributed that blind electroporation attempts are unlikely to succeed. In addition, the success rate of SCE is heavily dependent on micropipette tip shape. Optimization of tip shape requires a process of trial-and-error, which for DNA plasmid delivery cannot provide immediate reliable feedback until the next day when protein expression is (or is not) evident.

An alternative to SCE is bulk electroporation, which takes advantage of the same principles as SCE for delivery of genetic material into cells, but instead of delivering plasmid and current through the same pipette, it utilizes large plate electrodes that are positioned on opposite sides of the structure targeted for transfection and simple pressure injection to deliver plasmid into the extracellular space between the electrodes (Muramatsu et al., 1998; Falk et al., 2007). This method permits the efficient transfection of multiple plasmids or other charged materials just like SCE, but instead of targeting only one cell it is used to target many cells within larger tissue volumes. One common example of this technique is *in utero* electroporation, in which plasmid is injected into the brain ventricles of embryonic animals and electroporation pulses are delivered through forceps-like paddle electrodes that bracket the uterus to generate an electric field within the brain of the embryo (Tabata and Nakajima, 2001; Shimogori and Ogawa, 2008). The obvious advantage of this approach is that it does not require clear visualization or precise positioning of the electrode and is therefore applicable in nearly any tissue.

In the current paper, we describe CRE-Mediated Single-Cell Labeling by Electroporation (CREMSCLE), an innovative method that utilizes bulk electroporation to achieve the benefits of single-cell labeling for *in vivo* time-lapse imaging. CREMSCLE involves a binary co-expression approach that takes advantage of the ability of extremely low levels of Cre recombinase protein to edit many copies of a plasmid containing a neomycin “stop cassette” flanked by loxP sites that has been inserted into the 5′ end of the open reading frame of a gene of interest. This cre-mediated editing event effectively releases translation suppression of the downstream gene of interest. Using this binary approach, we show that co-electroporation of high concentrations of plasmid containing a gene of interest preceded by the stop cassette, together with extremely low amounts of plasmid encoding Cre recombinase, results in high levels of gene expression in very sparsely distributed individual cells, which constitutes ideal cell labeling conditions for live imaging. We previously published an application of this method to express EGFP in individual retinal ganglion cells in neonatal mouse eyes (Dhande et al., 2011). Here, using the *Xenopus* tadpole, which permits easy access for electroporation and *in vivo* visualization of fluorescent protein expression, we compare and contrast CREMSCLE with SCE and demonstrate its effectiveness for sparse co-expression of multiple gene products in the same cells.

## Materials and Methods

### Animal Breeding and Husbandry

All animal experiments were approved by the Montreal Neurological Institute (MNI) Animal Care Committee in accordance with the guidelines of the Canadian Council on Animal Care. Tadpoles were bred by HCG-induced mating of albino *Xenopus laevis* frogs (NASCO) in the MNI Animal Care Facility. Embryos were then reared with regular solution changes in bowls containing Modified Barth Solution with HEPES (MBS-H) buffer.

### Constructs

pCAG-Cre, pCALNL-EGFP, pCALNL-DsRed are a generous gift from T. Matsuda & C. L. Cepko and are currently available through Addgene (plasmids 13775, 13770, 13769). pEGFP-N1 was from Clontech. mCherry was a generous gift from Dr. Roger Tsien. All plasmids were grown in DH5a competent cells (Life Technologies) and purified using endotoxin-free maxiprep kits (Qiagen).

### Bulk Electroporation

Albino tadpoles at stage 44–46 according to the criteria of Nieuwkoop and Faber (1956) were used for electroporation. Animals were anesthetized by immersion in MS222 (0.02% in 0.1 × MBS-H). DNA solution containing various concentrations of either pEGFP-N1 or a mixture of pCAG-Cre with 1 μg/μL pCALNL-GFP and/or pCALNL-DsRed suspended in distilled water with a small amount of fast green dye for visualization was pressure injected in the brain ventricle with a glass micropipette (Borosilicate glass with filament, 1 mm OD, 0.78 mm ID, Sutter) pulled using a Sutter P-97 puller. Care was taken to load the ventricle without visibly distending it. Two custom-built platinum plate electrodes (cut to approximately 1 mm width strips from Sutter FB-330B platinum filaments) were placed on each side of the tectal lobe and current was applied using an electrical stimulator (SD 9, Grass Instruments), with a 3 μF capacitor connected in parallel. Two pulses of 37 V, 1.6 ms duration were applied in both directions. The animals were then placed in fresh rearing solution (MBS-H) and kept in bowls for at least 24 h before screening for EGFP expression under an epifluorescence microscope.

### Single-Cell Electroporation

Tadpoles at stage 42–44 were anesthetized in MS222 (0.02% in 0.1x MBS-H) and placed on a Kimwipe under a fluorescent microscope. A micropipette filled with pEGFP-N1 plasmid (1 μg/μL) was positioned within the cell body layer of the optic tectum to perform single cell electroporation of tectal cells (Haas et al., 2001; Bestman et al., 2006; Liu and Haas, 2011). A 1 s train of 1 ms pulses at 200 Hz was passed through the micropipette with an electrical stimulator (SD 9, Grass Instruments) and monitored by an oscilloscope (Tektronix). Pulse trains were repeated twice and each hemisphere of the tadpole optic tectum was electroporated at two sites to increase yield.

### Live Imaging

Animals were screened for *in vivo* imaging at 48 h after electroporation. Screened animals were anesthetized in MS222 (0.02% in 0.1x MBS-H), placed in a custom-made Sylgard chamber that fit the tadpole’s body and sealed under a cover glass. A custom built two-photon microscope was used for all live imaging experiments. The microscope consisted of a converted Fluoview FV300 confocal microscope mounted on a BX61WI base (Olympus, Japan) with external R3896 multialkali PMTs (Hamamatsu, Japan) for detection of emission signal. Red and green emission light was simultaneously collected after passing through a 565DCLPXR beam splitter with HQ525/50 and HQ607/45 filters specially blocked for two-photon excitation (Chroma Technology, Brattleboro, VT, United States). Excitation was provided either by a Maitai-BB or InSight X3 Ti:Sapphire femtosecond pulsed IR laser at 910 nm or 990 nm (Spectra Physics, Mountain View, CA, United States). Optical z-series were collected at 1 μm intervals using either a 60x 1.1 NA LUMFL or 60x 1.0 NA LUM Plan FL N water immersion objective (Olympus), or for larger field imaging at 3 μm intervals using a 20x 0.5NA U Plan FL N air objective. After imaging, the animals were placed in an isolated well that contained 0.1x MBS-H, and imaged every 24 h up to 5 days post-electroporation.

### Analysis

Two-photon image stacks of dendritic arbors were denoised using CANDLE non-local means denoising software implemented in MATLAB (Coupé et al., 2012). For daily imaging data, cells were reconstructed in 3D from z-series two-photon stacks using the autodepth feature of Imaris 6.0 (Bitplane). All data are expressed as mean ± SEM, and *n* values refer to the number of cells for the morphology experiments. Arbor size and branch tip number were analyzed by repeated-measures two-way ANOVA using Prism 7.0 software (Graphpad).

## Results

**FIGURE 1:**
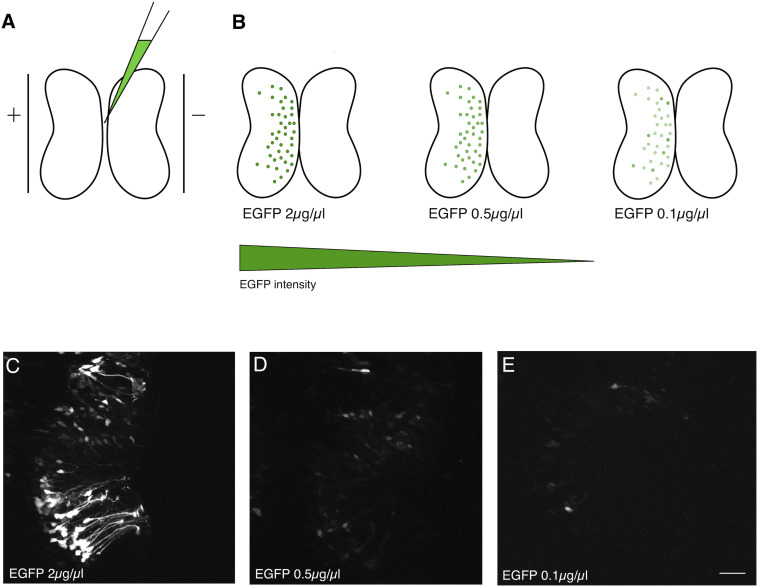
Dilution of electroporated plasmid reduces expression level. **(A)** Bulk electroporation of the Xenopus optic tectum involves the injection of a plasmid solution into the tectal ventricle followed by passing current pulses across plate electrodes that span the targeted brain region. Transduction occurs on the side toward the positive electrode due to the negative charge of plasmid DNA. **(B–E)** Reducing the concentration of EGFP plasmid mainly lowers the levels of expression per cell. **(C–E)** Two-photon z-series projections through the optic tectum of an intact Xenopus tadpole collected using the same laser excitation intensity. **(C)** At 2 μg/μL a large number of bright cells, especially radial glial progenitors, are labeled. **(D)** 0.5 μg/μL or **(E)** 0.1 μg/μL labels fewer cells, which are also much fainter. Scale bar, 50 μm.

Bulk electroporation of the optic tectum of *Xenopus* tadpoles can be performed by injecting a plasmid solution into the brain ventricle, and briefly passing current across a pair of platinum plate electrodes positioned on opposite sides of the brain (Figure 1A). Alternatively, the electrodes can be positioned over just one hemisphere. Since DNA is negatively charged, it will preferentially transfect the brain hemisphere closest to the positive electrode (Figure 1B). This method typically results in a large number of brain cells that express high levels of the gene product encoded by the electroporated plasmids (Figure 1C). In the case of EGFP plasmid electroporation, bright green cells are typically apparent within 24 h. The number of positive cells and apparent expression levels increase only incrementally over subsequent days.

In an effort to determine whether it would be possible to attain sparse EGFP expression, suitable for single-cell morphometric reconstruction, by simply reducing the concentration of plasmid injected, we compared tadpoles injected with a range of plasmid concentrations from 2 μg/μL to 0.1 μg/μL (Figure 1B). Although there was an apparent decrease in the number of transfected neurons expressing EGFP, the most obvious consequence of reducing plasmid concentration was that cells expressed lower levels of EGFP, making it difficult to visualize their full morphologies (Figures 1D,E). The cytomegalovirus immediate early (CMV) promoter used to drive EGFP expression on this plasmid is one of the strongest promoters available, suggesting that a strategy of simply trying different promoters would be unlikely to lead to enhanced brightness of EGFP fluorescence.

**FIGURE 2:**
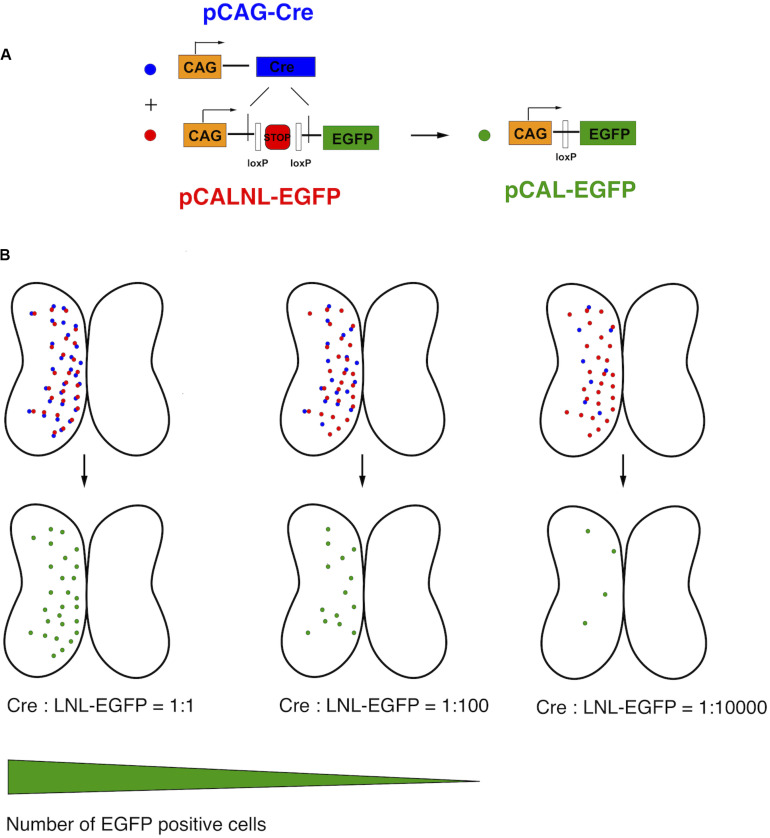
Schematic of CREMSCLE method. **(A)** Electroporation of large amounts of pCALNL-EGFP containing a floxed neogenin “stop cassette” does not lead to EGFP expression unless pCAG-Cre plasmid is co-expressed. The Cre recombinase removes the stop cassette flanked by loxP sites to allow the translation of EGFP. **(B)** When pCAG-Cre and pCALNL-EGFP are coexpressed in roughly equimolar ratios most cells will express EGFP. As the concentration of pCAG-Cre plasmid is reduced, keeping pCALNL-EGFP levels constant, only the very few cells that express Cre recombinase will activate pCALNL-EGFP to allow high levels of EGFP expression.

This relationship of plasmid concentration to EGFP expression level suggests that the brightly expressing cells are likely to be those that received a high number of copies of the plasmid. Therefore, we reasoned that high plasmid copy number contributed to bright EGFP expression. In order to express high amounts of EGFP in a very small number of cells, we devised a binary strategy in which the EGFP expression vector contained an upstream stop cassette, in this case part of a neomycin selectable marker, flanked by loxP sites and driven by the strong synthetic CAG promoter (pCALNL-EGFP). EGFP translation is thus prevented in all cells except those expressing both pCALNL-EGFP and a Cre recombinase vector (pCAG-Cre) (Figure 2A). Even very small amounts of Cre recombinase are able to effectively remove loxP-flanked sequences from a large number of plasmids, so the co-expression of very low concentrations of a plasmid expressing Cre recombinase together with high concentrations of LNL-EGFP plasmid ought to induce sparse but bright EGFP expression in the electroporated tissue (Figure 2B).

**FIGURE 3:**
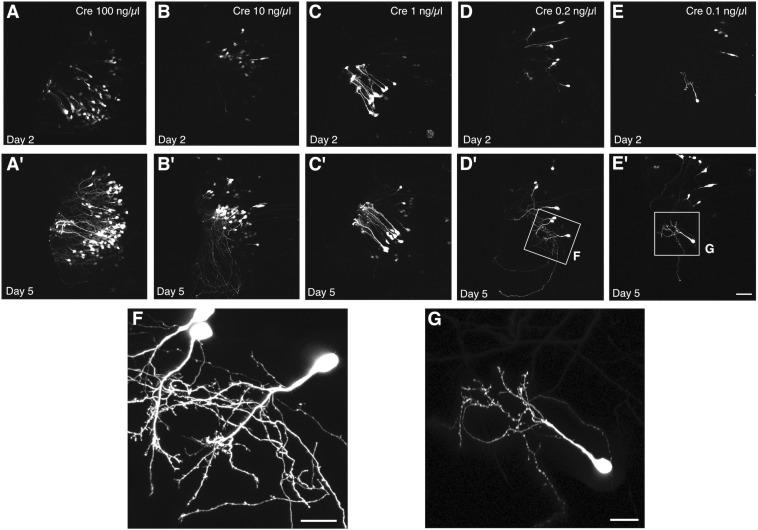
Titration of Cre permits single cell labeling without decreasing signal intensity. Two-photon z-projections of the transfected lobe of the tadpole optic tectum demonstrate that electroporation of 1 μg/μL pCALNL-EGFP plasmid together with increasingly dilute concentrations of pCAG-Cre plasmid results in a decreasing number of labeled tectal cells with little apparent decrease in the brightness of EGFP expression. Animals were imaged **(A–E)** 2 and **(A′–E′)** 5 days after electroporation with 1 μg/μL pCALNL-EGFP plus pCAG-Cre concentrations of **(A,A′)** 100 ng/μL, **(B,B′)** 10 ng/μL, **(C,C′)** 1 ng/μL, **(D,D′)** 0.2 ng/μL, or **(E,E′)** 0.1 ng/μL. **(F,G)** Cells continue to mature and develop complex dendritic arbors over this time as can be seen in higher magnification z-series projections. Only the lowest dilution of Cre plasmid produced cells with completely non-overlapping dendritic arbors by day 5, which would be suitable for single-cell reconstruction. Scale bar, **(A–E)** 50 μm, **(F,G)** 20 μm.

We used this co-electroporation strategy to label cells in tadpoles at developmental stage 44–46 with pCALNL-GFP at a concentration of 1 μg/μL, together with varying concentrations of pCAG-Cre (100, 10, 1, 0.2, and 0.1 ng/μL). At the highest concentrations of pCAG-Cre, a large number of cells with relatively simple but overlapping processes expressed EGFP in the optic tectum at 2 days post-electroporation (Figures 3A–C), including many radial glial cells (Figure 3C). Electroporations of lower concentrations of Cre recombinase plasmid, less than 1 ng/μL, clearly labeled individual cells with more easily discriminable processes (Figures 3D,E). Over subsequent days, expression levels increased, resulting in brighter labeling, but also a larger number of cells visibly expressing EGFP. By 5 days post-electroporation, dendritic processes had elaborated considerably in all cases (Figures 3A′–E′), but in the animals electroporated with less than 1 ng/μL of Cre plasmid, the low density of cells permitted clear visualization of the full morphologies of the labeled neurons and their dendrites at higher magnification (Figures 3F,G). In the example electroporated with the lowest concentration of Cre plasmid, 10,000 times more dilute than the pCALNL-EGFP, single cells were sparse enough to permit complete morphological segmentation without any overlap with another labeled cell (Figure 3G).

**FIGURE 4:**
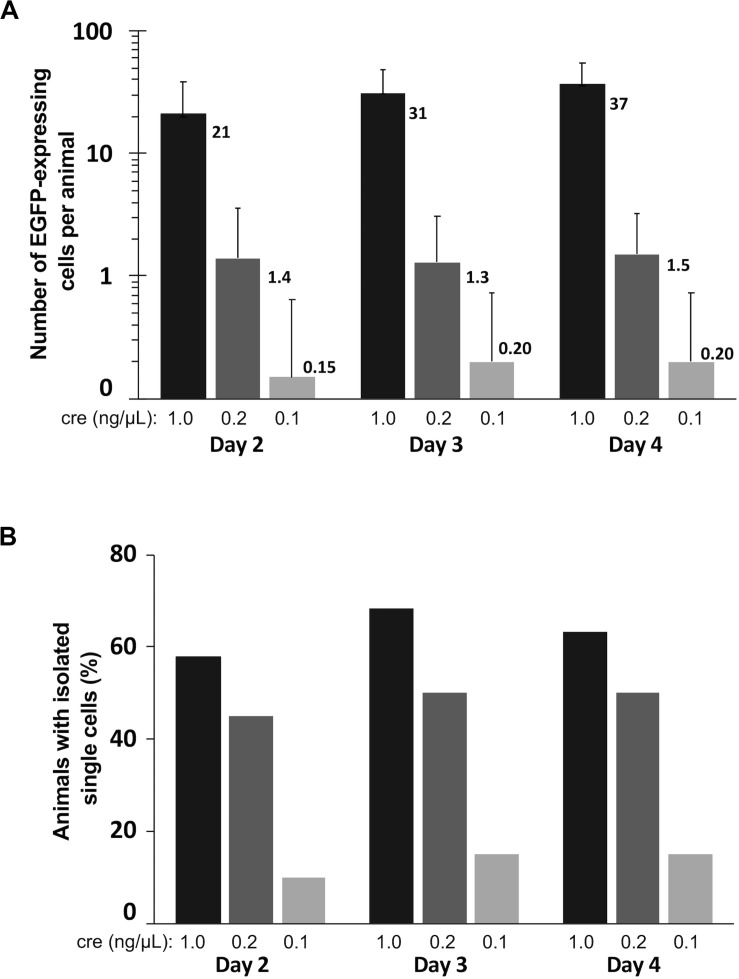
Quantification of labeled cells imaged over three consecutive days. Tadpoles were bilaterally electroporated with 1 μg/μL pCALNL-EGFP mixed with 1.0, 0.2, and 0.1 ng/μL pCAG-Cre plasmid (*n* = 19, 20, 20) and subsequently screened daily for EGFP-expressing cells from 2 to 4 days post-electroporation. **(A)** The number of EGFP-positive cells per animal was substantially lower with decreased levels of Cre plasmid. **(B)** A single isolated cell could be found in nearly half of the animals from the 1.0 and 0.2 ng/μL groups on all days of imaging, but much less frequently in the 0.1 ng/μL group. In this case the 0.2 ng/μL group had the most useful optimization, with sparse expression in a large proportion of animals (high-efficiency of single cell labeling).

To assess optimal concentrations of pCAG-Cre to co-electroporate with pCALNL-EGFP to label cells efficiently and at low enough densities for morphological characterization of individual cells, we performed bilateral electroporations in 60 animals divided evenly into three groups of 20 animals, each with 1 μg/μL pCALNL-EGFP plus a different concentration of Cre plasmid: 1, 0.2, and 0.1 ng/μL. All but one out of the 60 animals survived the electroporation procedure and recovered fully from anesthesia within a few minutes following the procedure. The number of EGFP-expressing cells per animal was quantified to assess density of labeling over 3 days of daily time-lapse imaging starting at 2 days post-electroporation (Figure 4A). The ideal case for morphometric analysis would be a single EGFP-labeled neuron per hemisphere (2 per animal). On average, 1 ng/μL of Cre plasmid resulted in well over 20 labeled cells per animal, which we found to be too high to reliably perform unambiguous single-cell morphological analysis. On the other hand, 0.2 ng/μL gave a much lower labeled cell density per animal, reaching 1.5 (range: 0–6) by 4 days post-electroporation, corresponding nearly to the ideal yield of one labeled cell per hemisphere on average. In these animals the cells started out relatively immature, with only 5.0 ± 5.0% of labeled cells per animal having a distinct neuronal morphology on day 2 after electroporation. But by day 3 and day 4, respectively, 41.1 ± 13.6% and 45.5 ± 13.9% of labeled cells had started to exhibit typical neuronal morphologies, with the remaining cells likely being radial glia and ependymocytes. The labeled cell density per animal dropped to 0.2 (range: 0–2) when the Cre plasmid concentration was halved to 0.1 ng/μL, reflecting a much lower probability that the animal had been successfully transfected to express EGFP. These findings demonstrate that with the appropriate ratio of Cre and reporter plasmid transfection, CREMSCLE has the potential to be a practical and highly efficient method for sparse labeling of cells.

We further quantified the proportion of animals under each of these conditions in which at least one isolated EGFP-expressing cell body, potentially suitable for subsequent reconstruction and quantitative analysis was present (Figure 4B). The efficiency of single-cell labeling was fairly low (15–20% of animals) when 0.1 ng/μL Cre plasmid was co-electroporated with LNL-EGFP. However, in the higher concentration cases, about half of the animals had at least one isolated, single cell present on day 4 (0.2 ng/μL:50%; 1 ng/μL:63% of animals). In this particular experiment, using a mix of 0.2 ng/μL pCAG-Cre with 1 μg/μL pCALNL-EGFP we were able to achieve both high numbers of labeled animals and sparse overall cell densities within each animal, which together are close to ideal for studying morphometry. This is imperative because even a well-isolated neuron can, over time, extend its dendritic processes into fields of labeled dendrites from other nearby cells, limiting its usefulness for quantitative reconstruction. Achieving this goal requires careful titration of the mix of Cre and LNL-EGFP expressing plasmid, depending upon the developmental stage of the animal and the efficiency of the specific set of electrodes used for electroporation, as small differences in the plate electrode shapes and the distance between electrode poles can alter transfection efficiency.

**FIGURE 5:**
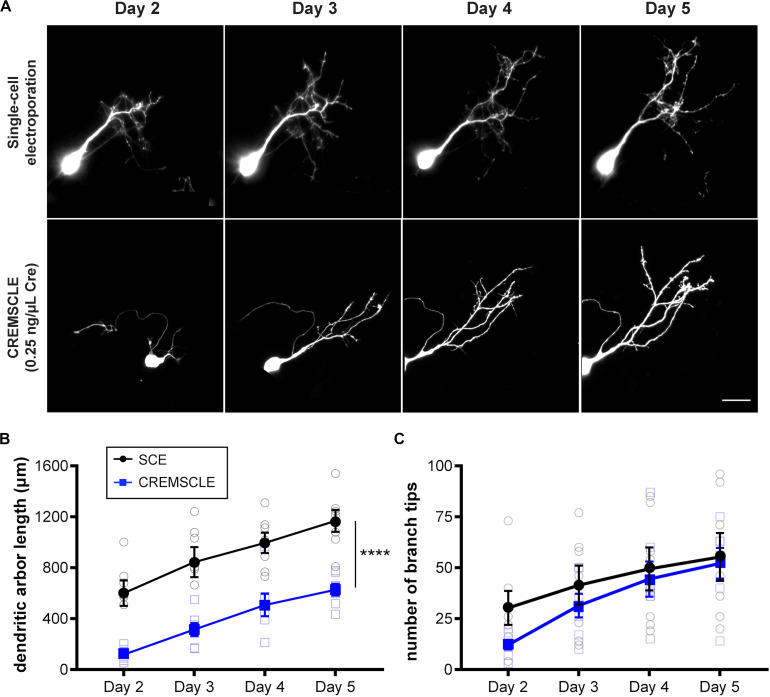
CREMSCLE tends to label more immature tectal neurons than SCE. Tadpoles were electroporated by SCE with pEGFP-N1 (1 μg/μL) or by CREMSCLE with pCAG-Cre (0.25 ng/μl) and pCALNL-EGFP (1 μg/μL), and subsequently imaged daily for EGFP-expressing cells from 2 to 5 days post-electroporation. **(A)** Two-photon z-projections of single cells following labeling by SCE or CREMSCLE. **(B,C)** Quantification of total dendritic arbor length **(B)** and number of dendritic branch tips **(C)** over 4 days of consecutive 2-photon imaging. Scale bar, 20 μm. ****p < 0.0001 for main effect, RM ANOVA, *n* = 7 cells per group.

It can be advantageous in certain studies, to be able to image very immature, recently differentiated neurons, and although this has been carried out with great skill using SCE (Hossain et al., 2012), it is straightforward with CREMSCLE. During electroporation of plasmids injected into the ventricular space, radial glia progenitors are more susceptible than postmitotic neurons to incorporating the plasmid, since they have their cell somata in the subependymal layer of the optic tectum in close proximity to the ventricle (Sharma and Cline, 2010). Differentiated neurons migrate away from the ventricular zone after they are born (Bestman et al., 2012). Consequently, bulk electroporation is more likely to target glia and immature newborn neurons than is SCE through a micropipette positioned in the cell body layer of the tectum. CREMSCLE is therefore a particularly useful method for following the life cycle of individual neurons from birth to full integration into the neuronal circuit. To ascertain whether more immature neurons are labeled using CREMSCLE than by SCE, we labeled single cells using both methods and performed daily two-photon imaging of their dendritic arbors over a 4 days period, starting from 2 days after electroporation (Figure 5A). Based on the empirically determined transfection efficiency with this set of electrodes, we decided to use pCAG-Cre at 0.25 ng/μL for this experiment. Two-photon z-series stacks of tectal neurons were used to manually reconstruct dendritic arbors in 3-dimensions for morphometric analysis. The dendritic arbor lengths of neurons labeled by CREMSCLE were significantly smaller than those labeled by SCE (*p* < 0.0001 repeated-measures ANOVA main effect, *n* = 7 cells per group), consistent with their being more immature (Figure 5B). Indeed, day 2 SCE-labeled cells appeared to be closest in size to day 5 CREMSCLE cells. Similarly, dendritic branch tip number also appeared to be slightly, but non-significantly, lower in the CREMSCLE neurons (Figure 5C). Robust EGFP expression in cells imaged a week or more after electroporation, suggests that the earlier developmental onset of expression did not limit subsequent imaging as the cells matured. These results indicate that CREMSCLE is suitable for labeling recently born immature neurons and following their development over time as they integrate into the circuit.

**FIGURE 6:**
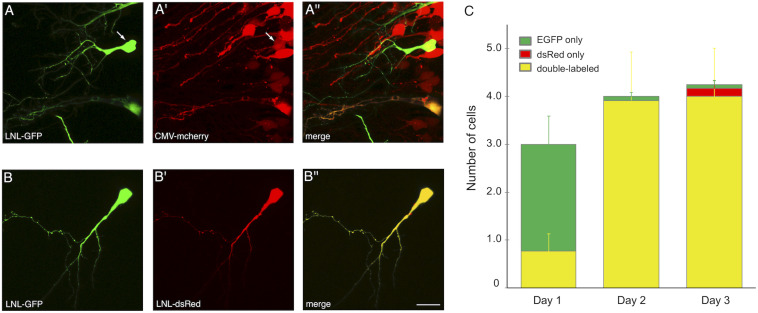
CREMSCLE can be used for co-expression of two proteins. **(A,B)** Two-photon z-projections of double labeled cells at 3 days post-electroporation. **(A)** pCAG-Cre (0.5 ng/μL) co-electroporated with pLNL-EGFP (1 μg/μL) and **(A′)** pCMV-mCherry (3 μg/μL) leads to **(A′′)** double labeling of sparse EGFP-expressing cells within a field of mCherry-labeled cells. Most cells labeled by EGFP are also positive for mCherry. **(B)** Co-electroporation of pCAG-Cre (0.1 ng/μL) with pLNL-EGFP (1 μg/μL) and **(B′)** pLNL-dsRed (3 μg/μL) results in nearly all cells expressing **(B′′)** both EGFP and dsRed by 3 days post-electroporation. **(C)** Number of cells per animal that expressed EGFP fluorescence (green), dsRed fluorescence (red), or both (yellow). By 2 days post-electroporation nearly every cell was double-labeled. *n* = 12 animals. Scale bar, 20 μm.

One of the strengths of SCE, for example over viral transduction, is the ability to co-transfect multiple plasmids into the same cell and achieve high probability of co-expression (Haas et al., 2001). We tested the CREMSCLE method to confirm that high rates of co-expression are also possible. It can be achieved either by simple bulk co-electroporation of a second expression vector (Figure 6A) or by placing the second gene of interest downstream of its own LNL stop cassette (Figure 6B). The first configuration is useful when it is desirable to densely express one of the two constructs in a large number of cells, for example to study the effects of intercellular signaling or competition for a secreted factor on single cell morphogenesis. In the second configuration, low-concentration Cre plasmid can activate both plasmids, resulting in a sparse distribution of predominantly double-labeled cells. To assess the efficiency of co-expression in sparsely labeled cells, we electroporated a mixture of 0.1 ng/μL pCAG-Cre with 1 μg/μL pLNL-EGFP and 3 μg/μL pLNL-dsRed, choosing a ratio of 1:3 in an effort to ensure that each EGFP-positive cell also expresses dsRed. On the first day following electroporation, each tadpole had an average of 3.0 ± 0.8 labeled cells, the majority (74%) of which appeared to exclusively have green fluorescence at this time point (Figure 6C). However, by the next day the number of labeled cells had increased to 4.0 ± 1.1 per animal, and 98% of the cells were now expressing both EGFP and dsRed. The perceived increase in double-labeling is likely due to the slower maturation rate of dsRed protein compared to EGFP (Baird et al., 2000). By day 3, a small number of cells appeared to exhibit exclusively green (2%) or red (4%) fluorescence emission though a majority of cells (94%) continued to clearly express both fluorophores. These results show a very high rate of double-labeling for the vast majority of cells, and many of these cells were well-isolated and bright enough to fully visualize dendritic morphology (Figure 6B).

## Discussion

We have presented a quantitative characterization of the CREMSCLE technique for sparse but bright labeling of single cells through the co-electroporation of extremely low concentrations of plasmid encoding Cre recombinase together with an EGFP or other expression vector into which a stop cassette has been inserted upstream of the open reading frame to inhibit protein translation in cells lacking Cre expression. This method, which benefits from the versatility and ease of bulk electroporation, provides bright labeling of sparsely distributed cells, which is an ideal condition for *in vivo* imaging of cellular morphology and time lapse imaging of axonal and dendritic branch remodeling. We first introduced this technique in a 2011 paper that examined the morphologies of individual retinal ganglion cell axon arbors in the thalamus and superior colliculus of the neonatal mouse (Dhande et al., 2011). The current study expands on that initial report with valuable technical details to help investigators select the right ratios of Cre to expression plasmid for efficient single cell imaging. Sparse labeling is particularly important to allow unambiguous discrimination of single neurons and their fine processes from the complex mesh of neuropil into which they are embedded. Furthermore, it facilitates time-lapse imaging over many days, during which time cell morphologies can change significantly, normally making it extremely difficult to find the same cell in a densely labeled field. We also demonstrate that CREMSCLE is particularly advantageous for visualizing stem cells, neuroblasts, and immature neurons in the brain. Finally, we show that double-labeling and co-expression of genes-of-interest occur with very high frequency using CREMSCLE.

CRE-mediated single-cell labeling by electroporation represents an effort to simplify and generalize the innovative methods originally developed for sparse labeling in transgenic mice, such as Mosaic Analysis with Double Markers (MADM; Zong et al., 2005) and Single-neuron Labeling with Inducible Cre-mediated Knockout (SLICK; Young et al., 2008). These powerful genetic approaches were limited by the substantial investment of time and resources needed to generate suitable transgenic animals, and the lack of availability of transgenic lines in non-model organisms. By relying entirely on electroporation for gene expression, CREMSCLE mitigates these limitations. Furthermore, because bench-ready electroporation constructs can be generated by simple subcloning of plasmids, any gene of interest can be prepared for CREMSCLE in just a matter of days, making it equally suitable for single-cell expression of fusion proteins for subcellular targeting, genetically encoded functional indicators like pHluorins, calcium- or voltage-indicators, as well as constructs for RNA interference (LoTurco et al., 2009; Sun et al., 2019).

The major advantage of CREMSCLE over juxtacellular labeling methods like SCE is the ability to get sparse gene expression in a large fraction of electroporated animals without the need to visualize the precise placement of an electroporation micropipette. Although sophisticated visualization techniques have been developed, such as “shadowpatching,” in which the extracellular space is filled with a fluorescent dye to reveal negative images of somata by two-photon imaging to guide electroporation or recording pipettes (Kitamura et al., 2008), this is time consuming and requires expensive hardware compared with the CREMSCLE technique. These issues can be circumvented by using a bulk electroporation approach. The inspiration for CREMSCLE came from a technique for temporal control of expression of bulk electroporated genes developed by Matsuda and Cepko (2007), which used co-electroporation of LNL constructs together with an estrogen-receptor-tagged (ER) Cre recombinase that requires tamoxifen to induce excision of the stop cassette and drive gene expression.

Bulk electroporation has become extremely popular in recent years due to its low cost, versatility and applicability across many animal models, including tadpole, chick, rat, mouse, and ferret (Fukuchi-Shimogori and Grove, 2001; Gal et al., 2006; Shimogori and Ogawa, 2008; LoTurco et al., 2009; Kawasaki et al., 2012). In particular, transfecting and labeling cortical excitatory neurons by *in utero* electroporation offers a high success rate and lamina specificity, exploiting the staggered birthdates and inside-out migration of neurons within the cortex. A recent study applied a variant of the CREMSCLE method for imaging single neurons in the mouse visual cortex labeled by *in utero* co-electroporation of plasmids for Cre recombinase and Cre-dependent flip-excision (FLEX) expression of fluorescent protein (Sun et al., 2019). The advantage of the FLEX approach is that because the gene-of-interest is double-floxed and inverted in the original vector, there is no possible way for faint, leaky expression of the gene to occur in the absence of Cre recombinase. Another clever approach for overcoming possible faint background expression of the silenced construct is the Supernova system, which utilizes a positive feedback loop to drive expression of both the gene-of-interest along with a tetracycline transactivator (Luo et al., 2016). The transactivator acts back on the tetracycline response element on the Cre plasmid to enhance Cre expression levels, activating further copies of the gene-of-interest.

In the current study, we used the strong CAG promoter to drive gene expression. Other studies have taken advantage of electroporation of constructs with cell-type specific promoters to target expression to different cell classes (Gal et al., 2006). In the Xenopus system we have observed that cell type specificity is often difficult to achieve by conventional bulk electroporation or SCE, most likely because the high plasmid copy number that must be delivered to achieve adequate *in vivo* visualization of cells may overwhelm endogenous promotor specificity. Although we have not systematically tested it, we predict that use of a cell-type specific promoter for the highly diluted Cre plasmid would be more likely to undergo normal promoter control and produce better cell type specificity. In addition to cell type specificity, better temporal specificity of sparse gene expression could also be achieved by using ER-Cre with tamoxifen dosing in CREMSCLE (Matsuda and Cepko, 2007; LoTurco et al., 2009).

One of the main shortcomings of the CREMSCLE method compared with SCE, concerns the co-electroporation of non-genetically encoded materials, such as antisense Morpholino oligonucleotide (MO) for knockdown of gene expression. When plasmid DNA and fluorescently tagged MO are delivered by SCE though the same micropipette, both materials are jointly targeted to a single cell with high probability, labeling the single cell that is subject to gene knockdown (Bestman et al., 2006). In contrast, bulk electroporation of MO causes knockdown in a large number of cells in the zone of electroporation, thus in combination with CREMSCLE knockdown in the labeled single cell is likely to occur, but will not be restricted to just the one cell, much like co-electroporation with an expression vector that does not contain a floxed stop cassette (Figure 6A). Because bulk electroporation efficiently targets stem cells in the ventricular zone, one area for future development could be the merging of CREMSCLE with genome editing approaches like Clustered Regularly Interspaced Short Palindromic Repeats (CRISPR) (Mikuni et al., 2016). This would allow for targeted gene knockout or tagging by homology-directed repair, which could potentially be achievable by including a floxed stop cassette in the co-electroporated Cas9 or single guide RNA expressing vectors.

## Data Availability Statement

The raw data supporting the conclusions of this article will be made available by the authors, without undue reservation.

## Ethics Statement

The animal study was reviewed and approved by the Montreal Neurological Institute Animal Care Committee.

## Author Contributions

ER conceived the experiments. AS performed the experiments, except for Figure 5, executed by ZC. All authors contributed to writing the manuscript.

## Conflict of Interest

The authors declare that the research was conducted in the absence of any commercial or financial relationships that could be construed as a potential conflict of interest.
